# Estimated Amounts of β-Carotene, Vitamin B6, Riboflavin and Niacin in the Daily Diet of Older Subjects Associate Negatively with ADP-Induced Aggregation of Blood Platelets Independently of Cardiovascular Risk Factors

**DOI:** 10.3390/nu17213461

**Published:** 2025-11-02

**Authors:** Kamil Karolczak, Agnieszka Guligowska, Bartłomiej K. Sołtysik, Joanna Kostanek, Tomasz Kostka, Cezary Watala

**Affiliations:** 1 Department of Haemostatic Disorders, Medical University of Lodz, ul. Mazowiecka 6/8, 92-215 Lodz, Poland; joanna.kostanek@stud.umed.lodz.pl (J.K.); cezary.watala@umed.lodz.pl (C.W.); 2 Department of Geriatrics, Healthy Aging Research Center (HARC), Medical University of Lodz, Pomorska 247/249, 92-209 Lodz, Poland; agnieszka.guligowska@umed.lodz.pl (A.G.); bartlomiej.soltysik@umed.lodz.pl (B.K.S.); tomasz.kostka@umed.lodz.pl (T.K.)

**Keywords:** blood platelets, platelet aggregation, arachidonic acid, collagen, adenosinediphosphate, ADP, aging, vitamin, diet

## Abstract

**Background/Objectives:** Platelet-dependent thrombotic risk increases with age. Little is known, however, about the potential effect of vitamins on platelet reactivity in older subjects. **Methods:** Therefore, in the present study we examined the dependencies of whole blood platelet aggregability (in response to arachidonic acid (AA), collagen (COL) or adenosinediphosphate (ADP), using impedance aggregometry) in older men and women (60–65 yr, *n* = 246) on the intakes of vitamins (vitamins A, E, C, B_6_, B_12_ and D, niacin, thiamine, riboflavin, retinol, β-carotene and folates) with a typical daily diet (vitamin contents estimated using Dieta 5.0 software). **Results:** Overall, significant negative bootstrap-boosted partial correlation coefficients, adjusted for selected cardiovascular risk factors, were revealed for AA and β-carotene, and ADP and β-carotene, riboflavin, vitamin B_6_ and niacin. These findings were further validated by the outcomes of the bootstrap-boosted canonical analysis, confirming the relationships revealed for ADP, and to a lesser extent for AA. COL-dependent platelet aggregation appeared to not be associated with the amount of vitamins in the subjects’ daily diet. **Conclusions:** Hence, we conclude that the intake of vitamins in the daily diet of older subjects is negatively associated with platelet aggregability in an agonist- and vitamin-specific manner.

## 1. Introduction

Previous research on the aging of the cardiovascular system allows us to identify several typical features of the heart and blood vessels characteristic of people of an advanced age. The features of an aging heart include an increased number of apoptotic and necrotic cells, a greater likelihood of developing fibrosis and the appearance of ischemic tissue. These changes can often lead to heart rhythm disturbances. Blood vessels can also be severely affected by the aging process and show increased myocyte infiltration, arterial thinning, decreased nitric oxide synthesis, fibrosis, arterial stiffening, ischemia and atherosclerotic plaque deposition [1]. The causes of the above changes should be investigated in the heart muscle itself and in the cells of the vascular walls. However, we should not forget about the other factors present in the circulatory system, which can modulate in a beneficial or unfavorable way almost all of the above-mentioned cardiovascular aging processes. And what could these factors be, at least hypothetically? The obvious clue seems to be blood platelets, the fragments of the cytoplasm of megakaryocytes evolved to interact with blood vessels through direct intercellular interactions or through factors secreted by platelets themselves [2,3].

Indeed, it seems that blood platelets can significantly affect the main aging-related features of the heart muscle and blood vessels. Changes in the expressions of membrane proteins and granule secretions from activated platelets may prevent heart tissue regeneration. For example, after hypoxia-induced heart damage the influx of mesenchymal cells to the heart may be inhibited by activated blood platelets [4]. Activated platelets are able to induce apoptosis in various types of cells of the cardiovascular system [5]. Cardiac fibrosis can be effectively inhibited using clopidogrel, a well-known antiplatelet drug [6], which suggests that platelet-dependent events are involved in heart fibrosis. The supporting evidence for this may be the observation that the successful inhibition of heart remodeling with antiplatelet drugs may result in a lower rate of arrhythmias [7]. It seems that overactivated platelets significantly contribute to the remodeling of blood vessel walls, favoring the influx of myocytes [8] and neointimal hyperplasia [9]. Thus, platelet activation contributes significantly to cardiovascular aging. It is desirable to keep the activation and reactivity of platelets at a low, physiological level, allowing them to maintain their hemostatic role while simultaneously reducing their involvement in pathological thrombosis.

In older people, however, it is not so easy to keep blood platelets at the physiological level of activation and to not overcome the red line of overactivation and increased reactivity. This may be due to the prolonged exposure of blood platelets to high levels of various atherogenic factors, including glucose, a condition often noticed in older people [10,11]. This, in turn, translates to a higher risk of platelet-dependent thrombosis [12].

Thus, platelet activation contributes significantly to cardiovascular aging. However, very little is known about the basic parameters of platelet reactivity, like their sensitivity to arachidonic acid, collagen or ADP and their readiness to aggregate after stimulation with platelet agonists. The direct measures of the functional state of blood platelets of older people remain unknown.

It is desirable to keep the activation and reactivity of platelets at low, physiological levels, allowing them to maintain their hemostatic role while reducing their involvement in pathological thrombosis. This can be achieved with antiplatelet drugs, but in some cases it is difficult to establish the proper pharmacological algorithm in older patients [13], since their blood platelets often show enhanced excitability in response to platelet activators and decreased sensitivity to inhibitors [14,15]. One of the approaches aiming to protect blood platelets against activating factors is the modulation of environmental factors, including diet.

The richness of the studies dealing with different foods and single molecules is enormous. Platelet reactivity can be reduced by dark chocolate, garlic, ginger, omega-3 PUFA, onion, purple grape juice, tomato and wine [16]. Despite the fact that it is difficult to unambiguously attribute the antiaggregatory effects to specific dietary components when considering the chemical complexity of the mentioned examples of ‘antiplatelet foods’, it is generally believed that polyphenols and fatty acids represent one of the strongest antiplatelet food components [17]. Some studies, however, associate a slight increase in collagen- and epinephrine-induced aggregation with the Mediterranean diet [18]. Even so, due to the great diversity of research methodologies and the lack of basic data on, inter alia, the bioavailability of dietary ingredients and their real concentrations in human tissues, it is still difficult to draw unequivocal conclusions from studies on the antiplatelet effects of dietary components [19].

The reactivity of blood platelets can be tracked with the support of impedance aggregometry, a method allowing the measurement of and commonly utilized to monitor thrombotic risk [20,21,22]. To trigger the aggregation of blood platelets in this approach, we employed arachidonic acid, collagen and adenosinediphosphate (ADP). Arachidonic acid and ADP after secretion from blood platelets can be found in the surrounding milieu of blood plasma, where they can autocrinally activate platelets [23]. Collagen, in turn, is the main subendothelial protein, which is exposed when the vascular wall is damaged and exhibits the greatest thrombogenic capacity [24,25]. Other platelet agonists represent plasma-soluble substances secreted by blood platelets into the extraplatelet environment, where they may exert an autocrine-activating effect on platelets via G protein-coupled (GPCRs) receptors [23].

Surprisingly, little is known about the possible effects of vitamin intake on the reactivity of blood platelets, with one particular exception, i.e., strong antioxidants such as vitamins C and E, which are the most exhaustively studied in terms of their impact on blood platelets [26,27,28,29,30]. Despite this, the effect of vitamins on the activation and reactivity of platelets in humans, particularly in the geriatric subpopulation, is poorly explored and recognized, especially in non-in vitro models. Thus, we are facing two research gaps: the rare involvement of older subjects in investigations on the effects of diet on platelet reactivity and scarce studies performed with the use of in vivo models.

Therefore, the aim of the present study was to examine the relationships between platelet reactivity (aggregability), measured as the platelet response to arachidonate, collagen or ADP stimulation, and the amount of a set of vitamins (vitamin A, retinol, alpha-tocopherol, thiamine, riboflavin, niacin, vitamin B_6_, vitamin C, folate, vitamin B_12_ and vitamin D) present in the daily diet of women and men aged 60–65 years.

Below we present three consistent lines of evidence arising from the outcomes of three types of statistical analyses.

## 2. Materials and Methods

### 2.1. Chemicals

Arachidonate, equine tendon collagen and ADP were purchased from Chrono-Log Corp. (Havertown, PA, USA). Physiological buffered saline (PBS) was obtained from Avantor Performance Materials Poland S.A. (Gliwice, Poland) and S-Monovette^®^ blood collection systems were from Sarstedt (Nümbrecht, Germany).

### 2.2. Study Design

The following results were obtained in the course of the project “The occurrence of oxidative stress and selected risk factors for cardiovascular risk and functional efficiency of older people in the context of workload”, funded by the Central Institute For Labour Protection—National Research Institute (Warsaw, Poland) and supervised by the Clinic of Geriatrics at the Medical University in Lodz (Poland). The two basic inclusion criteria comprised age within the range of 60 to 65 years, and the willingness to participate. The inclusion and exclusion criteria were identical to those described earlier [31].

The research group included roughly 300 subjects (equal sex proportions of 150 men and 150 women), aged from 60 to 65 years. The general characteristics of the studied group are presented in Table 1. After excluding volunteers taking antiplatelet drugs, 246 volunteers (124 men and 122 women) proceeded to the study.

The experiments reported here were undertaken in accordance with the guidelines of the 1975 Helsinki Declaration for Human Research. The study was approved by the Committee on the Ethics of Research in Human Experimentation, Medical University of Lodz. A written layout of the experiment with detailed information about its objectives, design, risks and benefits were presented to each of the participants before blood sampling. Informed written consent was obtained from each individual at the beginning of the experiment. After the acceptance of the plan of the project granted by the Committee on the Ethics of Research in Human Experimentation at the Medical University of Lodz in 2014, all the procedures involving volunteers (all steps of recruitment, blood sampling and measurements of platelet aggregation made on freshly isolated blood) were performed and finished in 2015.

### 2.3. Blood Sampling, Isolation of Blood Plasma, Measurements of Blood Morphology and Serum Biochemistry

Blood was taken after overnight fasting and 15 min rest directly before blood donation. Blood was collected by aspiration either to vacuum tubes (Sarstedt, Nümbrecht, Germany) supplemented with 0.105 mol/l buffered sodium citrate (citrate/blood ratio = 1:9, *v*/*v*) for platelet activation and reactivity measurements, to tubes coated with EDTAK_3_ for blood morphology analysis, or to tubes without any anticoagulant for further biochemical determinations. In all cases blood was collected from a peripheral vein cannulated with an 18-gauge needle.

Blood morphology was measured with a 5-Diff Sysmex XS-1000i hematological analyzer (Sysmex, Kobe, Japan), while serum biochemistry was evaluated with a DIRUI CS 400 analyzer (Dirui, Changchun, China). In order to obtain blood serum, whole blood was collected in the tubes without an anticoagulant, incubated for 30 min at 37 °C and centrifuged (2000× *g*/15 min./4 °C). The supernatant (serum) was aspirated and used in further analyses (basic blood serum biochemistry).

### 2.4. Whole Blood Impedance Aggregometry

Platelet aggregability was measured with the use of a MultiPlate Analyzer (Dynabayte, Munich, Germany), as described previously [15]. In brief, samples of citrated blood were left for 10 min for blood repositioning at 37 °C to avoid any artefactual platelet activation caused by aspiration. Then, 300 µL aliquots of whole blood were gently mixed with an equal volume of PBS and left at 37 °C for three minutes. Each sample was supplemented with either 0.5 mmol/L arachidonate, 1 µg/mL collagen or 10 µmol/l ADP to trigger platelet aggregation. The recording of platelet clumping started immediately thereafter and was tracked for at least 15 min.

Platelet aggregability was represented as the area under an aggregation curve (AUC) and the maximal aggregation (A_max_). These two variables give an optimal measure of the extent of platelet aggregation: how high they are when platelets respond maximally, and how long it lasts before disaggregation begins. These two measures combined together are much more reliable measures of the extent of platelet aggregation than just one of them, and it is more difficult to justify selecting one for the final description of aggregation. Therefore, a third variable combining the previous two was used to obtain a more complete description of platelet aggregation: the normal scores for each of the two variables were calculated (A_max_ and AUC) to provide a combined variable representing both measures; this was calculated as (AUCxA_max_)/1000, in order to use an integrative index combining the degree of maximum aggregation (Amax) and the “total” effect over time (AUC), with the scaling factor of 1000 added for clarity/numerical accuracy

The present study was based on impedance aggregometry of whole blood. This was an optimal choice for this study as it allows the fast processing of blood samples to avoid artefactual activation, which is often the case in platelet-rich plasma or isolated platelets in blood obtained from older subjects. Also, using samples of whole blood allows us to avoid the need for centrifugation, which can significantly affect basic platelet physiology.

Arachidonic acid, collagen and ADP were used to stimulate pathways known to induce platelet aggregation. The arachidonic acid-dependent pathway is a molecular target of a popular antiplatelet drug, acetylsalicylic acid [15], which also affects collagen [15]. ADP stimulates a pathway commonly targeted by thienopyridines [32].

Citrate was used as an anticoagulant to avoid fast, spontaneous artefactual platelet activation in vitro, which can occur when heparin or hirudin are used. The chance of in vitro artefactual platelet activation increases with the length of the procedure. Citrate considerably slows down the process and maintains the blood platelets in a near physiological state regarding their internal calcium pool; as such, citrate-anticoagulated platelets respond correctly to in vitro stimulation with agonists. EDTA was not used for anticoagulation as it can disturb platelet membrane proteins and result in pseudothromocytopenia. Citrate was a more suitable anticoagulant for the present study [33,34], as it prevents overwhelming artefactual activation, while maintaining platelets in a nearly physiological state; in addition, the baseline physiological state of the studied platelets was verified using flow cytometry.

### 2.5. Vitamin Intake

The daily vitamin intake was calculated based on a detailed analysis of the participants’ diet. The intake of food and beverages during the preceding day was estimated by qualified investigators (dietitian and nutritionist) on the basis of a 24 h recall questionnaire with a portion size album. This interview was conducted over three days, and the diet that was considered by the participants as the most representative of their typical diet was taken for further analysis.

To decrease the risk of errors from such dietary recall, participants were asked to prepare a list of food products (including snacks and beverages) they ate on the day before the appointment. Interviewers were instructed not to judge the diet of the respondents. An album of photographs of food products and dishes was used to determine how many grams the eaten portion was [35]. None of the study participants used protein supplements. The nutritional products declared by patients were analyzed with Dieta 5.0 software, the reference method for evaluating the nutritional value of diets in Poland, developed by the National Institute of Food and Nutrition (Warsaw, Poland), and based on the Polish Food Composition Database. Dieta 5.0 constitutes a specialized computational tool intended for the planning and assessment of dietary intake at both the individual and population levels. Furthermore, it facilitates the real-time analysis of dietary consumption patterns within studied population groups in the context of national dietary recommendations. The primary purpose of the software is to enable the precise estimation of the energy and nutrient content of diets, as well as the quantities and types of foods consumed, by comparing the calculated nutritional values against established dietary guidelines [36,37]. The amounts of the following vitamins were estimated: A (equivalent of retinol), retinol, β-carotene, E (equivalent of tocopherol), thiamine, riboflavin, niacin, B_6_, vitamin C, folates, B_12_ and D. The amount of vitamins [µg or mg] given represents their levels of consumption with the participants’ typical daily diet.

### 2.6. Statistical Analysis

Continuous data was presented as either the mean ± SD or the median with interquartile range (from lower [25%] to upper [75%] quartile). Categorical data was presented as percentages. Data normality was tested with the Shapiro–Wilk W test and the homogeneity of variance with Levene’s test. Where the data was found to have a normal distribution and homogeneous variance, the groups were compared with a non-paired *t*-Student’s test; otherwise, the Mann–Whitney U-test was used. The associations between variables were calculated as Spearman’s rank correlation coefficients or Pearson’s partial correlations.

To estimate partial correlation coefficients upon the adjustment for the sets of potentially confounding variables, we used multiple regression analysis. We employed the canonical analysis to associate two sets of variables: (1) the aggregation of blood platelets in response to either arachidonic acid, collagen or ADP (representing blood platelet function); (2) including the studied vitamins. This method gave us not only the canonical correlation coefficients, reflecting the significance of the relations between the two sets of variables, but also the factor loadings representing the contributions of specific vitamins in the shaping of the agonist-dependent platelet reactivity. When analyzing categorical data, we used the Yates-corrected chi-square test or the exact Fisher’s test. In all the multivariate analyses, we implemented the bootstrap-boosted versions of statistical tests (10,000 iterations), which minimized the risk of rejecting a null hypothesis by pure chance. Statistical analyses were performed with Statistica v.13 (Dell Inc., Tulsa, OH, USA), StatsDirect v.3.0.182 and R Package Software v. 4.4, using an algorithm for data resampling in multiple regression analysis written by one of the authors (J.K.).

## 3. Results

The basic characteristics of the studied group are summarized in Table 1.

### 3.1. Simple (Not Adjusted) Correlations Between Platelet Aggregability and Intake of Vitamins with the Daily Diet

In the first statistical approach, aiming to evaluate the correlations between platelet aggregability and the intake of vitamins with daily diet in older subjects, we calculated simple Spearman’s correlation coefficients with no adjustment for any confounding factors.

We found that the aggregation of blood platelets induced with arachidonic acid correlated negatively in a statistically significant manner with the amounts of vitamin A, retinol, thiamine, riboflavin, niacin, vitamin B_6_ and vitamin B_12_ included in the typical daily diet of the investigated older subjects (Table 2).

The amounts of retinol, riboflavin and vitamin B_12_ in the typical daily diet of seniors were inversely and significantly correlated with the aggregation of blood platelets triggered with collagen (Table 2).

The amounts of only two of the tested vitamins, i.e., riboflavin and vitamin B_6_, were significantly and negatively associated with the responsiveness of blood platelets to ADP (Table 2).

### 3.2. Partial Correlation Coefficients After the Adjustment for the Selected Cardiovascular Risk Factors

In the second statistical approach, aiming to evaluate the correlations between platelet aggregability and the intake of vitamins in the daily diet of older subjects, we calculated partial correlation coefficients with an adjustment for cardiovascular risk factors grouped in three separate sets.

The components of these sets are widely accepted as important factors in cardiovascular risk shaping. Both in sets I and II, we used indices of platelet morphology, since the main subject of the present analysis is platelet aggregability that is directly dependent on platelet number [38]. Moreover, some works suggested that indices of platelet morphology can be considered as markers of cardiovascular risk [39], but not undisputably [40].

Both sets I and II include standard biochemical markers of cardiovascular risk, which are routinely measured to estimate cardiovascular health. All of these factors, i.e., triglycerides, HDL, LDL, glucose and uric acid, can significantly affect platelet aggregability [41,42,43].

The main difference between set I and set II comes down to the numbers of red (RBCs) and white blood cells (WBCs), which are present in set I, but are absent in set II. This reflects the plausible participation of RBCs and WBCs in shaping cardiovascular risk and also platelet aggregability [44,45,46,47,48,49,50,51]. However, since these markers are not so routinely considered as indicators of cardiovascular health, we designed one set which is RBC/WBC (+) and another set, which is RBC/WBC(−).

Due to the fact that the main subject of current analyses is the amount of vitamins in diet, we created set III, which includes dietary factors, which are the most general indicators assessing the content of the three most basic classes of nutrients, i.e., proteins, lipids and carbohydrates.

Set I included sex, age, count of white blood cells (WBCs), count of red blood cells (RBCs), concentration of hemoglobin (HGB), haematocrit (HCT), count of blood platelets (PLTs), mean platelet volume (MPV), plateletcrit (PCT), platelet distribution width (PDW), platelet–large cells ratio (P-LCR), serum concentrations of triglycerides (TG), HDL (HDL), LDL (LDL), glucose (GLU) and uric acid (UA); set II comprised sex, age, PLT, MPV, PCT, PDW, P-LCR, TG, HDL, LDL, GLU and UA. We also performed the calculation with the adjustment for set III of the confounding factors, including sex, age, intake of protein, intake of carbohydrates, intake of fat with the subjects’ typical daily diet and the energy supplied from the subjects’ typical daily diet.

We have revealed that the associations between blood platelet reactivity in older subjects and the amounts of vitamins present in the subjects’ daily diet show agonist-specific relationships, as follows.

In the case of the arachidonic acid-induced aggregation of blood platelets, only the amounts of β-carotene were significantly and negatively associated with platelet reactivity, but merely after the adjustment for the second set of cardiovascular risk factors. For both the first and the second set of cardiovascular variables, the associations between platelet reactivity and the amount of β-carotene in the daily diet of older subjects remained at the threshold value of *p* = 0.05 for the first set of variables and beyond significance (*p* = 0.08) for the second set of adjusting variables (Table 3).

The amounts of vitamins present in the typical daily diet of older subjects remained not associated with the reactivity of blood platelets to collagen.

When ADP was used to trigger the aggregation of blood platelets, we observed that the amount of vitamin A, retinol, thiamine, vitamin C, folates, vitamin B_12_ and vitamin D did not show significant associations with platelet reactivity.

The amounts of β-carotene estimated in the typical daily diet of older men and women was significantly negatively associated with platelet reactivity to ADP after the adjustment for each of the three sets of confounding risk factors.

Moreover, statistically significant associations were found between platelet ADP-dependent aggregability and the amounts of riboflavin and vitamin B_6_ after the adjustment for the first and the second set of cardiovascular risk factors, but not after adjusting for set III.

Niacin was found to be significantly negatively associated with the ADP-induced aggregation of blood platelets only after the adjustment for the second set of cardiovascular risk factors. For this vitamin, we only revealed a statistical tendency (*p* = 0.05) in the case of the adjustment for the first set of cardiovascular risk factors and a definitive lack of statistical significance (*p* > 0.05) in the case of the adjustment for the third set of variables (Table 3).

Likewise, only statistical tendencies, but beyond statistical significance, were found for the associations of ADP-dependent platelet aggregation and amounts of vitamin E in the subjects’ typical daily diet after the adjustments for either the first (*p* = 0.06) or the second (*p* = 0.05) set of cardiovascular risk factors, with no significant association upon adjusting for the third set of factors (*p* > 0.05) (Table 3).

### 3.3. Canonical Correlations Between the Grouped Variable “Diet Vitamins” and Blood Platelet Aggregation in Older Subjects

In the next attempt, we employed canonical analysis to evaluate the potential contribution of the vitamins present in the typical daily diet of older subjects to the aggregability of blood platelets dependent on arachidonic acid, collagen or ADP. In this approach, we implemented the procedure of the adjustment of variables further employed in the generation of canonical functions for risk factors grouped into three sets according to the pattern described earlier. Thus, in the canonical analysis we evaluated the possible associations between the grouped variable “vitamins” including all of the vitamins estimated in the daily diet and the particular types of platelet aggregation, i.e., arachidonate- or collagen- or ADP-dependent platelet aggregation. This procedure was performed with the data subjected to prior adjustment for each of three groups of risk factors (set I, set II and set III) (see more detailed explanation in Section 2.6)”. After having employed the procedure of canonical analysis to the prepared data, we revealed an interesting pattern: statistically significant canonical correlations can be found for the arachidonate- and ADP-dependent aggregation of blood platelets, but not for collagen-induced platelet reactivity and this pattern exists regardless of whether the employed adjustment was performed for the first, the second or the third set of cardiovascular risk factors (Table 4).

Canonical analysis allowed us to determine the contributions of each vitamin in the shaping of the specific agonist-dependent variability of blood platelet aggregation. Among the tested vitamins, we found that for the arachidonate-dependent aggregation of blood platelets the most significant contributors were vitamin B_6_, vitamin E, thiamine, β-carotene, niacin and riboflavin. For the ADP-dependent aggregation of blood platelets, the most significant contributors were vitamin B6, riboflavin, niacin, thiamine, β-carotene, vitamin E and folates. Interestingly, in all of the variants, regardless of the set of cardiovascular risk factors used to adjust the revealed relationships, and also regardless of the type of platelet aggregation, vitamin B6 appeared to be a leading vitamin contributing to the association between platelet aggregability and the amounts of the vitamins in the subjects’ typical daily diet. Interestingly, the series of the other vitamins following vitamin B_6_ as the further most significant contributors to the associations between arachidonate- or ADP-dependent aggregability and the intake of vitamins by older subjects appeared quite constant and included thiamine, riboflavin, β-carotene and niacin (Table 4).

### 3.4. Correlations of Recommended Daily Allowance of Vitamins and Platelet Reactivity in Older Men and Woman

The collected dietary interviews allowed us also to estimate the RDA—an average daily dietary intake of vitamins sufficient to meet vitamin requirements (%RDA). We checked whether the %RDA of individual vitamins correlated with platelet aggregation dependent on arachidonic acid, collagen or ADP. For this purpose, we also used a multi-step statistical analysis: simple (unadjusted) correlations were calculated, then partial correlation coefficients were adjusted for the three sets of variables (set I—sex, age, count of white blood cells (WBCs), count of red blood cells (RBCs), concentration of hemoglobin (HGB), hematocrit (HCT), count of blood platelets (PLTs), mean platelet volume (MPV), plateletcrit (PCT), platelet distribution width (PDW), platelet–large cells ratio (P-LCR), serum concentrations of triglycerides (TG), HDL (HDL), LDL (LDL), glucose (GLU) and uric acid (UA); set II—sex, age, PLT, MPV, PCT, PDW, P-LCR, TG, HDL, LDL, GLU and UA; set III—sex, age, intake of protein, intake of carbohydrates, intake of fat with the subjects’ typical daily diet and the energy supplied with the subjects’ typical daily diet) and, finally, a canonical analysis was performed (also after adjustment for the three sets of cardiovascular variables (sets I, II and III identical to above).

A simple (not adjusted) Spearman’s rank correlation revealed that only the %RDA of two of the tested vitamins, i.e., riboflavin (R_s_ = −0.158) and vitamin B_12_ (R_s_ = −0.159) and collagen-dependent aggregation showed significant (*p* < 0.05 in both cases) and negative correlations.

Looking at the correlations between arachidonic acid-triggered platelet aggregation and the RDAs of vitamins, we found only one significant pair of associations, i.e., with the RDA of vitamin B_12_ (R_S_ = −0151; *p* < 0.05).

ADP-dependent platelet aggregation did not reveal itself as significantly correlated with the %RDA for any of the vitamins studied when simple (unadjusted) Spearman’s rank correlation coefficients were used for analysis.

Partial correlations showed that when cardiovascular variables from set I were used for adjustment, only ADP-dependent platelet aggregation, but not arachidonic acid- or collagen-induced aggregation, were significantly correlated with %RDA only for riboflavin (r_partial_= −0.148; *p* < 0.05) and for vitamin B_6_ (r_partial_= −0.136; *p* < 0.05). When cardiovascular variables from set II were used for adjustment, ADP-dependent platelet aggregation was again found to be significantly correlated with %RDA for riboflavin (r_partial_= −0.158; *p* < 0.05) and vitamin B_6_ (r_partial_= −0.146; *p* < 0.05), but moreover with %RDA for niacin (r_partial_= −0.133; *p* < 0.05). Platelet aggregation dependent on arachidonic acid and collagen was not found to be significantly correlated with %RDA for any of the vitamins studied when the second set of cardiovascular variables was used for adjustment in this statistical approach.

In the case of partial correlation coefficients adjusted for the third set of cardiovascular variables, no significant correlations were revealed between %RDA for any of the tested vitamins and any of the platelet reactivity types.

The canonical analysis revealed no statistically significant correlations for any of the studied RDAs and any of the markers of platelet aggregability (Table 5).

## 4. Discussion

Our findings indicate that a higher intake of some of the vitamins as part of the subjects’ daily diet is associated with a lower extent of platelet reactivity in older subjects, measured as the aggregation of blood platelets induced with either arachidonic acid, collagen or ADP.

We used three statistical approaches to estimate the potential relationships between the aggregability of blood platelets and the amounts of vitamin in the typical daily diet of older men and women. The first approach was a simple correlation of two variables with no adjustment to possible confounders. In the second approach, partial correlation coefficients were calculated, which allowed us to adjust these estimates for three different sets of potentially confounding risk factors. Two of these were referred to as typical (in a common epidemiological sense) cardiovascular risk factors, while the third included three markers representing the intakes of three major groups of nutrients: carbohydrates, proteins and lipids. This allowed us to see whether the associations between platelet aggregability and the intakes of vitamins with a typical daily diet by older subjects may be really shaped by selecting the analyzed confounders. Two sets of confounding risk factors (referred to as cardiovascular confounders) were arranged in such a way to include morphological and biochemical indices routinely measured during screening for cardiovascular risk, i.e., sex, age, the number of white blood cells, morphological markers of red blood cells and blood platelets, concentrations of HDL and LDL lipoproteins, triglycerides, glucose and uric acid. All of them have been recognized and indicated as being associated with the degree of platelet aggregation [41,50,52,53,54,55,56,57,58,59,60,61].

In the study presented herein, we employed a canonical analysis, including the statistical approach described above, in order to determine whether vitamins—as a group of molecules—show any correlations with platelet reactivity and, next, to put these vitamins in a descending order from the most to the least significant contribution to the noted association.

Among the tested vitamins we can found that two of them, i.e., vitamin C and vitamin D, appear to show no significant associations with any of the measured platelet aggregations (dependent on either arachidonic acid, collagen or ADP) in all three statistical approaches used in this study. Indeed, neither simple Spearman’s correlations nor the partial correlation coefficients, validated by the procedure of resampling with a replacement (10,000 iterations), appeared statistically significant. Also, based on the analysis of canonical correlation coefficients, we did not find vitamin C and vitamin D among the molecules significantly contributing to the associations between vitamins and platelet reactivity to arachidonic acid or ADP. Our results are in agreement with the previously published outcomes showing that vitamin C at low (0.3 mmol/L) or higher doses (3 mmol/L) is not able to affect platelet aggregation induced by ADP or collagen. The latter findings, however, were obtained with the use of isolated blood platelets prepared by a blood bank for transfusions, and therefore, should be interpreted more tentatively. Nevertheless, a very interesting observation from this study suggests that vitamin C can enter blood platelets and, hence that platelets can serve as a type of blood storage of vitamin C [30]; although, apparently, the storage of vitamin C in platelets is not related to any changes in platelet function. On the other hand, vitamin C deficiency has been found to be significantly related to cardiovascular events [62]. In this case, it is not surprising that vitamin C given orally at a dose of 1 g every eight hours for 10 days to patients with coronary artery diseases was found to significantly reduce platelet aggregate ratio [26]. Considering that in the subjects recruited to the present study, the median value of the daily intake of vitamin C was 77.3 mg with IQR 42.2–135.9 mg, we can easily find the potential reason for the discrepancy between the current results and those published earlier—the huge difference in the dosage of vitamin C. We can, thus, suggest that the significant antiplatelet effect of vitamin C is more likely to be observed with much higher doses than those present in the typical daily diet of the elderly subjects in this study. Moreover, only 47.4% of subjects in the current study reported a daily diet with a vitamin C content fully meeting the required daily intake.

Although the first reports on the role of vitamin A in cardiovascular health with an emphasis on blood platelets were published a century ago, no detailed investigation has yet been performed hitherto in this field. Early studies suggested that thrombocytopenia can be considered a reliable marker of vitamin A deficiency. However, while animals fed a diet deficient in vitamin A have been shown to experience decreased platelet counts, the authors regard this effect as insignificant and propose that thrombocytopenia cannot be regarded as a specific symptom of vitamin A deficiency [63].

Our knowledge on the role of vitamin A in cardiovascular health is very limited, but it has been proposed that vitamin A and its precursors can act as antihypertensive agents [64,65] and reduce atherogenic and cardiotoxic oxidative stress and inflammation [66,67,68]. While vitamin A may be considered a cardioprotective molecule, this conclusion still has a very limited experimental foundation.

The correlations found by us, especially in the more advanced statistical approach, i.e., after the implementation of the adjustment procedures and canonical analysis, do not support the view that the vitamin A present in the amounts estimated in the typical daily diet of the tested subjects can be considered as a significant factor shaping platelet aggregability in older subjects.

It has been found that retinol inhibits phospholipase A2 and thromboxane A2 synthesis [69]. Generally speaking, the ligands of the retinoid X receptor, including derivatives of vitamin A, such as 9-cis-retinoic acid, reduce the platelet response to thrombin and the agonists of GPVI, thus attenuating thrombus formation in a protein kinase A-dependent manner [70]. This may possibly explain why we observed a significantly negative correlation between the amounts of retinol in the subjects’ typical daily diet and platelet aggregability triggered by arachidonic acid or collagen. However, since these correlations were not confirmed by the outcomes of the analysis of canonical correlations and after the adjustment for cardiovascular risk factors, we believe, that at the current level of knowledge, retinol should not be regarded as a factor significantly influencing platelet reactivity in older subjects; however, since our results only comprise statistical coefficients of correlations, they are not valid as a reliable indicator of in vivo platelet reactivity in humans. Our results may only provide additional support for the idea that vitamin A may somehow influence platelet aggregation, but it is possible that this effect is also strongly modulated by another factor. This result allows us to formulate a hypothesis rather than draw definitive conclusions.

β-carotene, tested among other antioxidants, has been found to be generally inert to blood platelets and demonstrated no significant impact on blood platelets in young men and women (mean age of 31 years) after eight weeks of supplementation at 15 mg/day, despite a significant increase in its plasma concentration [71]. Our study used a completely different experimental design and methods, since we did not employ supplementation with β-carotene and we focused on older subjects. Despite the fact that β-carotene appeared as the vitamin with no significant simple relationships with any of the tested platelet reactivity types (the simple coefficients of Spearman’s correlation remained beyond significance, consistent with the report by Calzada et al. [71]), we were able to note significant coefficients of partial correlation after the adjustment for both sets of cardiovascular risk factors. Moreover, using this statistical method, we also found that ADP-dependent platelet reactivity is negatively and significantly correlated with the amount of β-carotene in the daily diet of older men and women, even after the adjustment for the variables of set III of the tested confounders, i.e., the amounts of proteins, carbohydrates and lipids in the subjects’ diet and the energy supplied by the subjects’ typical daily diet. We need to stress that the significant correlations between the ADP-dependent platelet aggregability and the subjects’ daily diet content of β-carotene, even after the adjustment for all three sets of the tested confounders, including the amounts of the major nutrients, appears quite unique among all other vitamins analyzed herein. Thus, β-carotene is outstanding in this view compared with all of the tested vitamins. Also, arachidonic acid-induced platelet aggregation seems to be affected by the amount of β-carotene in the subjects’ diet; but, this suggestion requires further study, since (after the adjustment for the amounts of proteins, carbohydrates, lipids and energy supplied by the subjects’ typical daily diet and also after the adjustment for the cardiovascular risk factors of set I) we detected that the statistical tendencies were still beyond statistical significance. The hypothesis on the antiplatelet action of β-carotene can be strengthened by our observation that this vitamin is always present in the group of vitamins that are the top candidates for the most significant vitamins contributing to the aggregability of blood platelets dependent on arachidonic acid or ADP. Hence, β-carotene seems to be a promising factor in possibly lowering platelet aggregability, even in the amounts present in the subjects’ daily diet (without additional supplementation).

Vitamin E is one of the best investigated antioxidant vitamins affecting blood platelets. Vitamin E intake has been associated with decreased platelet aggregation and secretion, and an increased sensitivity to inhibiting compounds [71]. However, it turns out that the antioxidant properties of vitamin E are not necessarily involved in its antiplatelet action [28] and it has only been evidenced that its main intraplatelet target is believed to be protein kinase C [72]. The strong antiplatelet effects of vitamin E announced in earlier studies are not so evident in the present study. We were able to detect only the statistical tendencies for the relationship of ADP-dependent platelet aggregation and the amounts of vitamin E upon the adjustment for sets I and II of cardiovascular risk factors. No significant relationships can be seen in simple Spearman’s correlations, but vitamin E is evidently involved in shaping arachidonic acid- and ADP-dependent platelet aggregability, as can be deduced from the outcomes of the canonical correlation analysis. Due to the differences in experimental approaches, it is difficult to combine the results of earlier studies dealing with the impact of vitamin E on blood platelets with the outcomes presented by us. But there are certainly some clues suggesting that, with more precisely and accurately designed experiments and statistical analysis, some interesting relationships can possibly be found.

Thiamine is involved in the regulation of the metabolism of carbohydrates and may be regarded as an anti-diabetic agent. It has been shown that 150–300 mg thiamine supplementation daily for one to three months can reduce fasting glycaemia levels and urinary albumin secretion [73,74,75]. Low thiamine levels have been reported in obese people with a BMI > 35 kg/m^2^ [76]. Thiamine can also decrease the levels of LDL [77]. Interestingly, lower thiamine concentrations were observed in people aged 50–70 with symptoms of depression—a very important risk factor for the further development of cardiovascular disease [78]. Thus, thiamine may have an important role in maintaining the cardiovascular health of older subjects [79]; however, results published to this date give no information on the possible direct impact of thiamine on blood platelets. Certainly, indirect effects can be suggested through lowering the levels of glucose or LDL. Our results show only the associations between the daily intake of thiamine and platelet aggregation and do not necessarily reflect direct thiamine–platelets interactions; but, importantly, statistical significance has been found merely in the case of simple Spearman’s correlations and exclusively for the aggregation of blood platelets induced with arachidonic acid. This association disappeared after the adjustment for all of the chosen cardiovascular risk factors. Nevertheless, the canonical analysis shows that thiamine can be placed among the top contributors shaping the relationship between arachidonic acid- and ADP-dependent aggregation and the amounts of vitamins in the typical daily diet of older subjects.

Riboflavin is another vitamin with a beneficial impact on cardiovascular risk profiles. It can reduce blood pressure in patients homozygous (TT genotype) for the 677C-->T MTHFR polymorphism, with higher homocysteine levels and with a lower rate of normalization of hypertension [80]. Hyperhomocysteinaemia can also be reduced by riboflavin treatment in older subjects [81] and it may be likely that this cardioprotective action may manifest by altering the metabolism of low-molecular-weight thiols; indeed, it has been found that cardiovascular mortality can be reduced significantly by riboflavin, but only in subjects with high blood levels of folates [82], another pivotal cofactor of enzymes metabolizing cysteine, homocysteine and glutathione. Since homocysteine is known as a significant coactivator of platelet reactivity [15,83], the observed inverse relationship between daily riboflavin intake and platelet reactivity may be in fact related to a reduction in homocysteine levels. However, the true cause–effect relations between riboflavin, homocysteine and platelet reactivity require further investigation.

Our current results do not yield any response to the question about the direct or indirect (through lowering homocysteine levels) action of riboflavin on blood platelets. Correlations found by us showed only a small reflection of the final effect, i.e., lower platelet aggregation when the intake of riboflavin in the subjects’ daily diet was higher. This was found in simple correlations regardless of the platelet agonist used but remained significant also after the adjustment for the cardiovascular risk factors only in the case of the ADP-induced aggregation of blood platelets. Interestingly, also in the case of ADP-dependent aggregability, the significant correlation with daily riboflavin intake disappeared after the adjustment for the daily intake of proteins, carbohydrates and lipids and the energy supplied by the subjects’ typical daily diet. Moreover, among the analyzed vitamins riboflavin appears as one of the most significant contributors to the relationships between daily vitamin intake and platelet reactivity calculated using canonical correlation. In this case, riboflavin is always in the sixth or third position among vitamins shaping the arachidonate- and ADP-dependent reactivity of blood platelets, respectively. Thus, despite the lack of any data on the exact mechanism of the antiplatelet action of riboflavin on the basis of the results published previously and also announced herein, it can be indicated as a strong candidate for an antiplatelet vitamin.

Niacin reduced the in vitro aggregation of blood platelets following ADP or COL induction. These changes were paralleled by the increased formation of prostaglandins D2, E2 and thromboxane B2; however, no changes in the expression of certain platelet reactivity and activation markers were noted, such as P-selectin, CD63, CD107a, CD154, CD165 or GPIIb/IIIa [84]. These changes evoked by niacin may be plausibly responsible for the negative associations observed by us between the intake of niacin with the subjects’ daily diet and platelet reactivity to arachidonate marked by simple Spearman’s rank correlation coefficients. In the case of the ADP-induced aggregation of blood platelets, we did not note a statistical significance, but a statistical tendency could be found. After the adjustment for the cardiovascular risk factors, we were no longer able to see a significant association between niacin intake and arachidonate-induced aggregation, what suggests that these potential associations are strongly impacted by those confounding factors. In turn, however, for the ADP-induced reactivity of platelets we have noted statistically significant associations with daily niacin intake, even after the adjustment for cardiovascular risk factors, but not for the main groups of nutrients (proteins, carbohydrates and lipids). Additionally, the loading factors in the canonical correlations revealed that niacin is always among the top vitamins contributing to the overall impact of vitamins on the ADP-dependent aggregation of older subjects. It can be suggested that niacin can be indicated as a potentially important vitamin associated with platelet reactivity in older subjects, with a more probable distinct association with the ADP-dependent platelet pathway, a less probable relation to the reaction dependent on arachidonic acid and no acknowledged correlation with the collagen-dependent platelet reactivity.

Earlier in vitro studies demonstrated the antiplatelet action of vitamin B_6_. Nevertheless, vitamin B_6_ acted as an antiplatelet agent most effectively at high, non-physiological concentrations [85,86]. It has been proposed that in order to achieve the significant inhibition of platelet aggregation by vitamin B_6_ in vivo, it should not be used alone, as a single vitamin, but rather as a mixture or complex of different vitamins [87]. In our study, vitamin B_6_ appeared as being negatively associated with platelet aggregability with arachidonic acid or ADP, but not with collagen, which were used as blood platelet agonists (simple coefficients of Spearman’s correlation). After the adjustment for cardiovascular risk factors and the main groups of nutrients (proteins, fat and carbohydrates) and energy intake, the significant correlations disappeared in the case of arachidonic acid-induced aggregation. Interestingly, the negative correlation between the ADP-triggered aggregability of blood platelets and the amount of vitamin B_6_ in the subjects’ typical daily diet remained significant even after adjusting for the cardiovascular risk factors (both sets), but not after the adjustment for the major set of intakes of nutrients and energy. Thus, the association of the dietary level of vitamin B_6_ with platelet aggregability in response to ADP is independent of morphological and biochemical cardiovascular risk factors but it is dependent on the daily intake of proteins, carbohydrates and lipids and the energy supplied by the subjects’ typical daily diet. Interestingly, this observation remains true for the amounts of vitamin B_6_ with no additional supplementation, i.e., for its amounts simply found in a typical daily diet and despite the fact that only half of our subjects took the required daily amount of this vitamin; thus, in the case of a much more balanced diet, the negative correlation between the amount of vitamin B6 and platelet aggregability could possibly be even greater. The finding remains in some agreement with earlier published reports [85,86]. Interestingly, in the canonical analysis of all of the tested variants, vitamin B_6_ was a leading vitamin contributing to the overall action of vitamins as the factor influencing platelet aggregability to ADP and arachidonate, which also confirms the results suggesting that the greatest inhibitory action of vitamin B_6_ on platelet aggregation can be achieved when it is used as a component of multivitamin mixtures [87].

The antiplatelet action of vitamin B_6_ may be associated with the induction of prostaglandin E1 synthesis, but may be independent of nitric oxide or prostaglandin D_2_ synthesis [87]. Therefore, it deserves further special attention as a potential inhibitor of platelet reactivity in older subjects, especially with regard to reactivity dependent on ADP. It is probable that vitamin B_6_ can support the antiplatelet action of thienopyridines or may be able to reduce the resistance of blood platelets to thienopyridines. Such a possibility should certainly be tested in further experiments.

Vitamin B_12_ is thought to correct cardiovascular risk through a reduction in the levels of homocysteine, a known coactivator of blood platelets [88]. Vitamin B_12_ was found to be significantly and negatively associated with the aggregation of blood platelets dependent on arachidonic acid and collagen when an analysis of simple Spearman’s correlation was performed. There was, however, no significant contribution of vitamin B_12_ to either of the platelet reactivities tested in the current study by the means of adjusted partial correlations or canonical analysis.

Studies involving folate supplementation in humans [89] and experimental folate deficiency [90] showed that folate is an efficient inhibitor of platelet activation and reactivity. It is strongly suggested that the observed antiplatelet effects of folate are caused by the folate-dependent reduction in hyperhomocysteinaemia [89]. It is difficult to compare our present results to those published earlier, which involved animal models or were focused on the presence of hyperhomocysteinaemia and supplementation with folic acid. Nevertheless, we revealed no significant correlations between the amounts of folates in the typical daily diet of the older subjects and platelet reactivity This outcome appears quite obvious, since the daily intake of folates in the studied group was extremely low and very much below the requirements.

Folates appeared to be one of the vitamins contributing to ADP-dependent platelet aggregation in the analysis of canonical correlations, but neither simple Spearman’s correlations nor partial correlations adjusted for additional risk factors revealed any significant associations between folates and either of the tested aggregations of blood platelets.

Several studies have assessed the relationship between vitamin D and platelet activation markers. In pregnant women with gestational diabetes and in Korean adults, a negative relationship was reported between the mean MPV parameter and the blood concentrations of vitamin D3 [91]. Interestingly however, in some other subgroups of patients significant correlations between vitamin D levels and the morphological markers of platelet activation were not found [92]. Vitamin D supplementation in people with type 2 diabetes (60,000 IU cholecalciferol/week for the first 3 months and 60,000 IU for a further 3 months as a maintaining dose) significantly reduced the level of platelet activation and oxidative stress [93]. It is also suggested that a larger amount of vitamin D in the blood may reduce the number of circulating platelet–leukocyte complexes [94]. Vitamin D has also been indicated as a factor that reduces the reactivity of platelets to collagen in patients with type 2 diabetes mellitus [95]. Thus, based on this random data, vitamin D might be perceived as a significant factor reducing platelet activation and reactivity. In our measurements, vitamin D remained completely unrelated to the degree of platelet aggregability. However, a few differences in the experimental approaches should be noted. We worked with blood platelets obtained from individuals representing quite a limited range of ages (60–65 years), while in some other studies the age range was much wider (for example 35–65 yr [93]). In addition, in other studies some very specific conditions were investigated, like type 2 diabetes mellitus [93,95] or a special supplement was implemented [93]. None of the previous studies worked with the amounts of vitamin D estimated in the subjects’ typical daily diet in the design accepted in our current study. This approach did not drive us to reveal any significant relationships between vitamin D and platelet aggregability in older subjects.

For the majority of the tested vitamins, we found that only about half of the men and women involved in the study achieved 100% or more than 100% coverage of the recommended daily allowances of vitamins. The best in this respect was the daily intake of riboflavin, vitamin B_12_ and niacin, for which 69.2, 54.2 and 53.4% of the surveyed volunteers, respectively, fully reached the recommended daily intake with their diet. For riboflavin, vitamin B_12_ and niacin, we also found high RDA values. Interestingly, these vitamins were noted as negatively and significantly associated with the reactivity of platelets in the different statistical approaches used by us in the current study. In the simple (not adjusted) analysis of Spearman’s correlations and in the analysis of partial correlation coefficients (where niacin and riboflavin were significantly and negatively associated with ADP-dependent platelet aggregation after adjustment for set I and II or the cardiovascular risk factors), riboflavin and niacin appear as one of the best contributors to the variable explaining arachidonic acid- and ADP-dependent platelet aggregability, also after adjustment for cardiovascular risk factors. In turn, folate and vitamin D, showing the lowest daily intakes (the lowest percentage of subjects with RDAs of at least 100% and also the lowest values of RDA), were found to have no significant relationship with the platelet reactivity parameters we tested here; however, few other vitamins taken in agreement with the recommended daily intake by around 50% of the tested subjects were also found to have no relationship to platelet reactivity. Generally, the statistical relationships found by us between the daily vitamin intake and platelet reactivity are rather modest, which may possibly be related to the quite low amounts of vitamins in the subjects’ daily diet. It seems that our results may be influenced by two main factors: the content of individual vitamins in the subjects’ diets (the degree to which the recommended daily requirement is covered) and specific types of interactions between individual vitamins and blood platelets. The latter interactions can be suggested to be highly dependent on the pathway of platelet activation triggered by different agonists (arachidonic acid versus collagen versus ADP). Looking at additional dietetic parameters like RDA values and the percentage of subjects with RDAs at least 100%, we can suggest that riboflavin, vitamin B_6_ and also niacin intake at appropriate levels may potentially lead to the most significant antiplatelet effects. We may also suggest that closer attention should be paid to the daily intake of folate and vitamin D in older adults and a further re-examination of platelet reactivity in this age group should be performed when an adequate daily intake of these two vitamins will be ensured.

The present study has some disadvantages, which should be taken into account when interpreting its findings. This study presents only the estimated amounts of vitamins present in the subjects’ typical daily diet, and not strictly the directly measured ones, and uses exclusively an analysis of statistical correlations, which indicates some possible associations, but may not necessarily reflect any cause–effect relationships. The implementation of more advanced statistical methods was designed to at least partially overcome these limitations through the presentation of the particular vitamins and platelet aggregations independent of some potential confounders, including cardiovascular risk factors, the intake of proteins, carbohydrates and lipids and the energy supplied by the subjects’ typical daily diet. Thus, we analyzed the correlations between each of the particular vitamins and each of the platelet reactivations (reflecting different—from a biochemical point of view—although partially overlapping, pathways of platelet aggregation) by taking into account the fact that both platelet aggregability and vitamins constitute a part of a wider constellation of cardiovascular risk factors, and not any isolated system per se.

Thus, the limitations of this study include the indirect assessment of vitamin content in the subjects’ daily diet, with no direct measurement in the body. Since such an estimation relies on interviews, it cannot be fully accurate. Moreover, since we presented only statistical correlations, the direct impact of particular vitamins on the platelet aggregation of older subjects still remains unknown. Although our study results also suggest that platelet aggregation should probably be lower with a higher daily vitamin intake, we are of course aware of the serious problem of vitamin bioavailability. Even if we accurately estimated the amount of vitamins in our daily diet, it does not necessarily mean that the entire amount becomes available to the cells. Since the number of factors affecting nutrient bioavailability is enormous, we had to, unfortunately, neglect this problem in our analysis. Regrettably, we were not able to perform our diet analysis over a longer period of time or to fully control variations in food composition.

We also attempted to rank the tested vitamins in the order from the most to the least significant contributors to platelet aggregability, using the calculation of factor loadings in the canonical analysis correlation. We performed the latter to highlight the few vitamins with the most probable clinical significance and to indicate those which raise the greatest hopes for the future. We emphasize that our study involved older participants, a group of subjects often misrepresented in studies who are especially worth a closer look and greater attention due to their higher thrombotic risk [12] and, commonly, accompanying problems with diet fortification with the required amounts of some vitamins [96].

In future, it is certainly desirable to make direct measurements of vitamin concentrations in the blood and relate it to platelet aggregability and other markers of platelet activation and reactivity in order to obtain direct measures of the relationships suggested by us. Also, in vitro studies on the effects of particular vitamins on blood platelets would be helpful to re-evaluate the above-presented associations. Since it is highly probable that vitamin intake is season-dependent (i.e., the higher availability of fresh fruits and vegetables during spring/summer and the solar-dependent synthesis of vitamin D), a longitudinal study would be optimal to give the most accurate results. We strongly suggest that blood platelets of older subjects should be more widely used in nutritional studies in further future study.

## 5. Conclusions

The intake of vitamins through diet is related to the degree of platelet aggregability in older subjects. The relationships found in this study are agonist-specific. It seems that the aggregation of blood platelets induced by arachidonic acid and ADP are particularly prone to the inhibitory action of some vitamins, whereas the aggregation of blood platelets dependent on collagen seems relatively insensitive to vitamins. Vitamin B_6_, β-carotene, riboflavin and niacin in relation to ADP-dependent aggregation deserve special attention as potential antiplatelet factors, as shown by the results of this study’s analysis of correlations with adjustment for cardiovascular risk factors. Also, AA-dependent aggregation may be considered to be negatively associated with the daily dietary amount of β-carotene. The role of the remaining vitamins in platelet aggregability cannot be entirely excluded but is more disputable and requires further verification. Due to the scarcity of data, it is necessary to design further in vitro and in vivo studies evaluating the role of vitamins in shaping platelet aggregation in older subjects.

## Figures and Tables

**Table 1:** Blood morphology, plasma/serum biochemistry, vitamin intake, anthropometric status, medical history and medical treatment variables reported in the studied individuals.

Variable	Both Sexes (*n* = 246)	Men (*n* = 124)	Women (*n* = 122)
*Indices of blood morphology and basic biochemistry*			
WBC (10^3^/mm^3^)	5.8 (5.0–6.9)	6.0 (5.0–6.9)	5.6 (5.07–6.8) ^U,^*
RBC (10^6^/mm^3^)	4.5 ± 0.4	4.7 (4.4–4.9)	4.3 ± 0.3 ^T,††^
HGB (g/dL)	13.8 (13.0–14.6)	14.4 (13.7–14.9)	13.3 ± 0.80 ^U,††^
HCT (%)	39.8 (37.6–41.6)	41.1 (39.2–42.6)	38.5 ± 2.2 ^U,††^
PLT (10^3^/mm^3^)	213 (181–243)	197 (168–228)	226.0 ±44.7 ^U,††^
MPV (µm^3^)	11.3 (10.8–12.1)	11.2 ± 0.9	11.35 ± 1.01
PCT (%)	0.24 (0.21–0.28)	0.22 (0.2–0.23)	0.26 ± 0.05 ^U,††^
PDW (fl)	13.6 (12.4–15.6)	13.5 (12.1–15.2)	13.8 (12.7–16.3) ^U,^*
P-LCR (%)	36.1 ± 7.7	35.7 ± 7.4	36.5 ± 8.4 ^T,^*
Lym (10^3^/mm^3^)	2.0 (1.6–2.4)	1.96 (1.5–2.2)	1.94 ± 0.5
Mono (10^3^/mm^3^)	0.5 (0.5–0.7)	0.57 (0.5–0.7)	0.51 (0.4–0.6) ^U,††^
Neu (10^3^/mm^3^)	3.1 (2.6–3.9)	3.18 (2.5–3.8)	2.99 (2.5–4.0)
Eo (10^3^/mm^3^)	0.2 (0.1–0.2)	0.13 (0.1–0.2)	0.17 (0.09–0.2) ^U,^*
Baso (10^3^/mm^3^)	0.03 (0.02–0.03)	0.03 (0.02–0.03)	0.03 (0.02–0.03)
Total cholesterol (mg/dL)	206.8 (173.8–237.3)	187.2 (168–218.3)	223 ± 49 ^U,††^
Triglycerides (mg/dL)	111.2 (76.8–161.1)	111.2 (77–141.3)	110.5 (78–164)
HDL (mg/dL)	48.4 (41.0–59.3)	44.3 (40–51.0)	54.25 (44–63) ^U,††^
LDL (mg/dL)	131.2 (103.4–156.5)	116.3 (101–139)	140 ± 39 ^U,†^
Glucose (mg/dL)	99.2 (91–108)	101.0 (93–111)	96.35 (89–105) ^U,#^
Uric acid (mg/dL)	4.84 ± 1.24	5.40 (4.8–6.1)	4.30 (3.8–5.2) ^T,††^
*Blood platelet reactivity*			
(AUCxA_max_)/1000_AA	319 (224–417)	291 (186–369)	354 (256–449) ^U,†^
(AUCxA_max_)/1000_COL	438 (328–587)	386 (287–535)	473 (356–645) ^U,#^
(AUCxA_max_)/1000_ADP	284 (206–376)	245.2 (170–343.)	322 (254 (408) ^U,†^
*Vitamin daily intake*			
Vitamin A (equivalent of retinol) [µg]	816 (521–1323)	849.9 (545–1330)	772 (465–1320) ^U,n.s.^
Retinol [µg]	275 (165–459)	343.3 (198–590)	224 (124–351) ^U,†^
β-carotene [µg]	2869 (1338–4692)	2732 (1278–3970)	2946 (1392–5053) ^U,n.s.^
Vitamin E (equivalent of alpha-tocopherol) [mg]	7.4 (5.1–11.5)	7.6 (5.3–12.4)	6.7 (4.4–10.1) ^U,n.s.^
Thiamine [mg]	1.1 (0.8–1.6)	1.3 (0.9–1.8)	0.9 (0.6–1.4) ^U,†^
Riboflavin [mg]	1.4 (1.1–1.9)	1.6 (1.1–1.2)	1.4 (1.1.–1.7) ^U,n.s.^
Niacin [mg]	15.5 (10.9–22.0)	17.9 (12–24)	14.0 (9.3–20.1) ^U,n.s.^
Vitamin B_6_ [mg]	1.7 (1.2–2.3)	1.8 (1.3–2.5)	1.5 (1.1–2.0) ^U,n.s.^
Vitamin C [mg]	77.3 (42.2–135.9)	67.3 (35–135)	83.9 (51–136) ^U,n.s.^
Folates [µg]	239.6 (180.1–312.6)	255.2 (188.2–333.4)	231.3 (172.0–292.8)
Vitamin B_12_ [µg]	2.6 (1.6–3.7)	2.8 (1.6–3.7)	2.1 (1.1–3.4) ^U,n.s.^
Vitamin D [µg]	1.8 (1.0–3.0)	2.1 (1.1–3.4)	1.6 (0.8–2.5) ^U,n.s.^
*Average recommended dietary allowances (RDAs) [%]*			
Vitamin A (equivalent of retinol)	104.4 (63.6–167.8)	94.0 (60.4–157.7)	109.1 (65.5–186.9)
Thiamine	93.1 (65.6–131.2)	98.1 (67.8–140.4)	86.5 (61.7–126.8) ^U,^*
Riboflavin	124.7 (92.3–154.9)	119.3 (87.9–153.1)	126.6 (101.8–154.9)
Niacin	105.2 (72.9–144.8)	111.1 (80.3–150.4)	98.9 (66.4–142.9) ^U,^*
Vitamin B_6_	104.1 (76.8–140.3)	105.1 (79.8–143.8)	97.5 (71.9–133.3)
Vitamin C	95.7 (50.1–175.2)	74.8 (39.6–150.3)	112.4 (70.3–186.5) ^U,^*
Folates	59.9 (45.1–77.9)	63.8 (47.9–84.2)	57.8 (43.0–73.0)
Vitamin B_12_	109.5 (67.6–154.6)	120.5 (66.9–155.8)	105.9 (68.3–151.3)
Vitamin D	12.1. (6.9–20.4)	14.2 (7.4–23.2)	11.1 (5.8–16.4)
*Percentage of subjects meeting the recommended daily intake [%]*			
Vitamin A (equivalent of retinol)	52.2	46.8	58.2
Thiamine	44.1	47.6	40.1
Riboflavin	69.2	62.1	77.1
Niacin	53.4	58.1	49.2
Vitamin B_6_	54.2	60.5	48.4
Vitamin C	47.4	37.9	57.4
Folates	13.4	16.1	10.7
Vitamin B_12_	57.1	62.1	52.5
Vitamin D	1.2	1.6	0.8
*Dietary indices*			
Daily energy intake [kcal]	1648.4 (1304.4–2221.5)	1930.5 (1491.7–2552.5)	1436.2 (1108.6–1893.2) ^U,n.s.^
Daily protein intake [g]	72.1 (54.9–85.8)	77.3 (59.8–94.0)	67.1 (50.0–81.4) ^U,†^
Daily fat intake [g]	56.5 (39.8–83.0)	65.0 (46.2–95.4)	50.1 (60.0–66.9) ^U,††^
Daily carbohydrates intake [g]	224 (172.0–293.0)	252.0 (194.0–322.0)	199.0 (152.0–264.0) ^U,††^
*Medical indices*			
Age [years]	62.4 ± 1.7	62.9 ± 1.7	62.6 ± 1.6 ^T,^*
Regular physical activity [%]	70	70	70
Current smoking [%]	23	24	22
BMI [kg/m^2^]	27.3 (24.7–30.3)	27.5 (25.06–30.4)	27.2 (24.4–30.04)
WHR	0.92 (0.84–1)	0.99 (0.94–1.04)	0.84 (0.80–0.91) ^††^
Hypertension [%]	47	54	39
Hypercholesterolemia [%]	63	59	66
Type 2 diabetes mellitus [%]	9	11	7
Myocardial infarction in the past [%]	2	2	0,8
Stroke in the past [%]	2	3	2
Osteoporosis [%]	11	2	20
Diseases of stomach and duodenum [%]	33	27	39
Cancer in the past [%]	7	6	7
Ophthalmology diseases [%]	20	19	20
Depression [%]	15	10	20
Chronic obstructive pulmonary disease [%]	13	11	14
Joints diseases [%]	48	44	52
*Medicines taken currently*			
Blocker of histamine receptor H2 [%]	0	0	0
Acetylsalicylic acid [%]	0	0	0
Clopidogrel or ticlopidine	0	0	0
Acenonocoumarol [%]	0	0	0
Nitrates [%]	0.4	0.8	0
Beta blockers [%]	19.5	19	20
Digoxin [%]	0	0	0
Angiotensin converting enzyme inhibitors [%]	18.2	18	19
Calcium channel blockers [%]	10.6	14	7
Indapamide [%]	11	13	9
Spironolactone [%]	0.8	0.8	0.8
Sartans [%]	5.7	5	6.5
Thiazide [%]	3.2	2.4	4
Amiloride [%]	0.4	0	0.8
Torsemide [%]	0.4	0	0.8
Eplerenone [%]	0	0	0
Alpha blockers [%]	5.7	11.3	0
Statins [%]	17	19	15
Fibrates [%]	2.4	0.8	4
Bisphosphonates [%]	2.4	0	5
Allopurinol [%]	2.4	3.2	1.6
Insulin [%]	2.4	5	0
Metformin [%]	6.5	6.5	6.6
Gliclazide/glimepiride [%]	3.7	5.6	1.6
Steroids [%]	1.6	0	3.3
Methotrexate [%]	1.2	0.8	1.6
Non-steroidal anti-inflammatory drugs [%]	2	2.4	1.6
Beta mimetics [%]	3.2	2.4	4
Antihistamines [%]	1.6	0	3.2
Antidepressants [%]	3.2	1.6	5
Neuroleptics [%]	0.8	0.8	0.8
Vinpocetine/nootropics [%]	3.7	4	3.3
Trimetazidine [%]	0.8	0.8	0.8
Mesalazine [%]	0.4	0	0.8
Trimebutine [%]	0.8	0.8	0.8
Diosmin [%]	2.4	0	5
Levodopa [%]	0.4	0.8	0

Variables (not adjusted) presented as means ± SD, median with interquartile ranges (from lower [25%] to upper [75%] quartile) or percentage fractions of a whole group of investigated patients. The amounts of the consumed vitamins ([mg]) represent the levels of consumption with a typical daily diet (without supplements). Comparisons between men and women were performed with an unpaired Student’s *t* test (^T^) or Mann–Whitney’s *U* test (^U^). * *p* ≤ 0.05, ^#^ *p* < 0.01, ^†^ *p* < 0.001, ^††^ *p* < 0.0001, ^n.s.^ *p* > 0.05. Abbreviations used: AA, arachidonic acid; ADP, adenosine diphosphate; A_max_, value of maximal platelet aggregation; AUC, area under aggregation curve; Baso, number of basophils; BMI, body mass index; COL, collagen; Eo, number of eosinophils; glycine; HCT, haematocrit; HDL, high-density lipoproteins; HGB, concentration of hemoglobin; LDL, low-density lipoproteins; Lym, number of lymphocytes; Mono, number of monocytes; MPV, mean platelet volume; Neu, number of neutrophils; PCT, plateletcrit; PDW, platelet distribution width; P-LCR, platelet–large cells ratio; PLT, platelet count; RBC, red blood cell count; WBC, white blood cell count; WHR, waist–hip ratio.

**Table 2:** Associations between the amounts of vitamins present in the subjects’ typical daily diet and the aggregability of blood platelets in older subjects (men and women).

	Arachidonic Acid	Collagen	ADP
Vitamin A [µg]	−0.134 ^#^	−0.074 ^n.s.^	−0.122 *
Retinol [µg]	−0.161 ^#^	−0.140 ^#^	−0.113 *
β-carotene [µg]	−0.079 ^n.s.^	−0.037 ^n.s.^	−0.103 ^n.s.^
Vitamin E [mg]	−0.122 *	0.012 ^n.s.^	−0.117 *
Thiamine [mg]	−0.126 ^#^	−0.099 ^n.s.^	−0.124 *
Riboflavin [mg]	−0.154 ^#^	−0.201 ^##^	−0.144 *
Niacin [mg]	−0.147 ^#^	−0.063 ^n.s.^	−0.120 *
Vitamin B_6_ [mg]	−0.149 ^#^	−0.089 ^n.s.^	−0.150 ^#^
Vitamin C [mg]	0.040 ^n.s.^	−0.053 ^n.s.^	−0.065 ^n.s.^
Folates [µg]	−0.063 ^n.s.^	−0.106 *	−0.056 ^n.s.^
Vitamin B_12_ [µg]	−0.148 *	−0.157 ^#^	−0.120 *
Vitamin D [µg]	−0.124 *	−0.113 *	−0.092 ^n.s.^

Analysis of the correlation between vitamin intake and platelet reactivity to arachidonic acid, collagen or ADP. The results are shown as Spearman’s rank correlation coefficients. Blood platelet reactivity was measured with impedance aggregometry (see Section 2) in response to agonists and recorded as area under aggregation curve (AUC) or maximal aggregation (A_max_). These variables were used to calculate (AUCxA_max_)/1000. The amounts of the consumed vitamins ([mg]) represent the levels of consumption (without supplements) in the subjects’ typical daily diet (for details see the Section 2 The amounts of vitamin A and vitamin E were estimated, respectively, as the equivalents of retinol and α-tocopherol. The coefficients of correlations with a statistical significance of *p* < 0.05 or *p* < 0.01 are indicated with the symbols of ^#^ or ^##^, respectively. The statistical tendencies (*p* = 0.05 or 0.05 < *p* < 0.1) are indicated with *; the coefficients that are statistically insignificant are marked as ‘n.s.’.

**Table 3:** Associations between the amounts of vitamins present in the subjects’ daily diet and the variables describing the aggregability of blood platelets in older subjects (men and women) after the adjustment for cardiovascular risk factors.

	Arachidonic Acid	Collagen	ADP
Vitamin A [µg]	−0.035 ^n.s.,I^ −0.046 ^n.s.,II^ −0.002 ^n.s.,III^	−0.053 ^n.s.,I^ −0.064 ^n.s.,II^ −0.067 ^n.s.,III^	−0.019 ^n.s.,I^ −0.072 ^n.s.,II^ −0.063 ^n.s.,III^
Retinol [µg]	−0.017 ^n.s.,I^ −0.028 ^n.s.,II^ 0.018 ^n.s.,III^	−0.044 ^n.s.,I^ −0.054 ^n.s.,II^ −0.060 ^n.s.,III^	−0.081 ^n.s.,I^ −0.090 ^n.s.,II^ −0.041 ^n.s.,III^
β-carotene [µg]	−0.128 *^,I^ −0.134 ^#,II^ −0.112 ^*,III^	−0.065 ^n.s.,I^ −0.068 ^n.s.,II^ −0.050 ^n.s.,III^	−0.167 ^#,I^ −0.172 ^##,II^ −0.157 ^##,III^
Vitamin E [mg]	−0.088 ^n.s.,I^ −0.103 ^n.s.,II^ −0.038 ^n.s.,III^	−0.028 ^n.s.,I^ −0.028 ^n.s.,II^ 0.009 ^n.s.,III^	−0.124 *^,I^ −0.129 *^,II^ −0.075 ^n.s.,III^
Thiamine [mg]	−0.037 ^n.s.,I^ −0.050 ^n.s.,II^ 0.052 ^n.s.,III^	−0.044 ^n.s.,I^ −0.056 ^n.s.,II^ −0.015 ^n.s.,III^	−0.068 ^n.s.,I^ −0.077 ^n.s.,II^ 0.009 ^n.s.,III^
Riboflavin [mg]	−0.074 ^n.s.,I^ −0.085 ^n.s.,II^ 0.017 ^n.s.,III^	−0.063 ^n.s.,I^ −0.080 ^n.s.,II^ −0.072 ^n.s.,III^	−0.156 ^#,I^ −0.169 ^#,II^ −0.060 ^n.s.,III^
Niacin [mg]	−0.080 ^n.s.,I^ −0.097 ^n.s.,II^ −0.004 ^n.s.,III^	−0.021 ^n.s.,I^ −0.038 ^n.s.,II^ −0.006 ^n.s.,III^	−0.126 *^,I^ −0.135 ^#,II^ −0.043 ^n.s.,III^
Vitamin B_6_ [mg]	−0.084 ^n.s.,I^ −0.103 ^n.s.,II^ −0.026 ^n.s.,III^	−0.060 ^n.s.,I^ −0.081 ^n.s.,II^ −0.056 ^n.s.,III^	−0.142 ^#,I^ −0.162 ^#,II^ −0.084 ^n.s.,III^
Vitamin C [mg]	−0.026 ^n.s.,I^ 0.023 ^n.s.,II^ 0.025 ^n.s.,III^	−0.072 ^n.s.,I^ −0.074 ^n.s.,II^ −0.069 ^n.s.,III^	−0.070 ^n.s.,I^ −0.071 ^n.s.,II^ −0.086 ^n.s.,III^
Folates [µg]	−0.000 ^n.s.,I^ −0.010 ^n.s.,II^ 0.045 ^n.s.,III^	−0.060 ^n.s.,I^ −0.075 ^n.s.,II^ −0.070 ^n.s.,III^	−0.108 ^n.s.,I^ −0.116 *^,II^ −0.043 ^n.s.,III^
Vitamin B_12_ [µg]	−0.007 ^n.s.,I^ −0.019 ^n.s.,II^ 0.045 ^n.s.,III^	−0.048 ^n.s.,I^ −0.058 ^n.s.,II^ −0.057 ^n.s.,III^	−0.104 ^n.s.,I^ −0.110 *^,II^ −0.040 ^n.s.,III^
Vitamin D [µg]	−0.054 ^n.s.,I^ −0.053 ^n.s.,II^ 0.035 ^n.s.,III^	−0.058 ^n.s.,I^ −0.057 ^n.s.,II^ −0.011 ^n.s.,III^	−0.060 ^n.s.,I^ −0.059 ^n.s.,II^ 0.022 ^n.s.,III^

Results are shown as the bootstrap-boosted partial correlation coefficients, estimated by the procedure of resampling with a replacement (10,000 iterations), after the adjustment for the variables included in each of the following three sets of confounding variables: set I—sex, age, count of white blood cells (WBCs), count of red blood cells (RBCs), concentration of hemoglobin (HGB), haematocrit (HCT), count of blood platelets (PLTs), mean platelet volume (MPV), plateletcrit (PCT), platelet distribution width (PDW), platelet–large cells ratio (P-LCR), serum concentrations of triglycerides (TG), HDL (HDL), LDL (LDL), glucose (GLU) and uric acid (UA); set II—sex, age, PLT, MPV, PCT, PDW, P-LCR, TG, HDL, LDL, GLU and UA; set III—sex, age, intake of protein, intake of carbohydrates, intake of fat with the subjects’ typical daily diet and the energy supplied by the subjects’ typical daily diet. Reactivity of blood platelets was measured with impedance aggregometry (see Section 2) in response to arachidonic acid (AA), collagen (COL) or ADP (ADP) and recorded as the index (AUCxA_max_)/1000. The amounts of the consumed vitamins ([mg]) represent the levels of vitamins consumed with the subjects’ typical daily diet (without supplements) (for details see Section 2). The coefficients of correlations with a statistical significance of *p* < 0.05 or *p* < 0.01 are indicated with the symbols ^#^ or ^##^, respectively. The statistical tendencies (*p* = 0.05 or 0.05 < *p* < 0.1) are indicated with *; the coefficients that are statistically insignificant are marked as ‘n.s.’.

**Table 4:** Canonical correlations between the grouped variable “diet vitamins” and blood platelet aggregation in older men and women.

Platelet Aggregation (Dependent Variable)	Total Redundance [%]	Explanatory Variables (Set 2)	Extracted Variance [%]	Total Redundance [%]	Canonical Correlation	Canonical Determination [R2]	p	Wilks’ Lambda	Best Contributors Expl Var
**set I**									
*AA-dependent*	9.667%	*vitamins*	10.689%	1.051%	0.307	0.094	0.021	0.903	Vit. B_6_, vit. E, thiamine, β-carotene, niacin, riboflavin
*collagen-dependent*	5.923%	*vitamins*	16.265%	0.963%	0.236	0.056	0.275	0.941	Vit. B_6_, riboflavin, thiamine, folates, vit. C
*ADP-dependent*	10.271%	*vitamins*	21.786%	2.281%	0.316	0.100	0.013	0.897	Vit. B_6_, riboflavin, niacin, thiamine, β-carotene, vit. E, folates
**set II**									
*AA-dependent*	9.723%	*vitamins*	10.774%	1.068%	0.308	0.095	0.020	0.903	Vit. B_6_, vit. E, thiamine, β-carotene, niacin, riboflavin
*collagen-dependent*	5.963%	*vitamins*	16.342%	0.973%	0.237	0.056	0.269	0.940	Vit. B_6_, riboflavin, thiamine, folates, vit. C
*ADP-dependent*	10.318%	*vitamins*	21.919%	2.301%	0.317	0.101	0.012	0.897	Vit. B_6_, riboflavin, niacin, thiamine, β-carotene, vit. E, folates
**set III**									
*AA-dependent*	9.718%	*vitamins*	10.751%	1.065%	0.308	0.095	0.020	0.903	Vit. B_6_, vit. E, thiamine, β-carotene, niacin, riboflavin
*collagen-dependent*	5.991%	*vitamins*	16.211%	0.969%	0.238	0.057	0.265	0.940	Vit. B_6_, riboflavin, thiamine, folates, vit. C
*ADP-dependent*	10.243%	*vitamins*	21.860%	2.281%	0.316	0.100	0.013	0.898	Vit. B_6_, riboflavin, niacin, thiamine, β-carotene, vit. E, folates

R^2^ is a squared canonical correlation coefficient (canonical determination), which is relevant to overall variance between canonical variables. Total redundancy is relevant to the variance between the canonical variable for set II (vitamins of the study) and the variable describing whole blood platelet response to the agonization with AA, collagen or ADP; it shows the representation of a given canonical variable by the explanatory variables of set II. Extracted variance is the variance between a given canonical variable and the variables that build it up; it reflects how well a given canonical variable represents a given set of variables. Wilks’ lambda is a parameter reflecting the contribution of set II to the explaining of the variance of set II; the lower the Wilks’ lambda, the higher the contribution. Canonical correlation was calculated with the use of the bootstrap-boosted procedure of resampling with replacement (10,000 iterations). The following vitamins were included in set II (vitamins): vitamin A, retinol, β-carotene, vitamin E, thiamine, riboflavin, niacin, vitamin B_6_, vitamin C, folates, vitamin B_12_ and vitamin D.

**Table 5:** Canonical correlations between the recommended dietary allowances of vitamins and blood platelet aggregation in older men and women.

Platelet Aggregation (Dependent Variable)	Total Redundance [%]	Explanatory Variables (Set 2)	Extracted Variance [%]	Total Redundance [%]	Canonical Correlation	Canonical Determination [R2]	p	Wilks’ Lambda	Best Contributors Expl Var
**set I**									
*AA-dependent*	6.460%	*vitamins*	12.880%	0.845%	0.249	0.062	0.183	0.935	Vit. B_6_, niacin, riboflavin, thiamine
*collagen-dependent*	4.539%	*vitamins*	18.536%	0.851%	0.207	0.043	0.398	0.955	Folates, vit. B_6_, riboflavin, vitamin C, thiamine
*ADP-dependent*	5.703%	*vitamins*	25.960%	1.538%	0.234	0.055	0.238	0.943	Vit. B_6_, niacin, riboflavin, thiamine
**set II**									
*AA-dependent*	6.441%	*vitamins*	12.776%	0.833%	0.248	0.062	0.186	0.936	Vit. B_6_, niacin, riboflavin, thiamine
*collagen-dependent*	4.491%	*vitamins*	18.535%	0.836%	0.206	0.042	0.403	0.955	Folates, vit. B_6_, riboflavin, vit. C, thiamine
*ADP-dependent*	5.681%	*vitamins*	25.921%	1.530%	0.233	0.054	0.240	0.943	Vit. B_6_, niacin riboflavin, folates
**set III**									
*AA-dependent*	6.740%	*vitamins*	12.102%	0.823%	0.254	0.065	0.158	0.933	Vit. B_6_, niacin, riboflavin, thiamine
*collagen-dependent*	4.519%	*vitamins*	18.791%	0.858%	0.206	0.043	0.396	0.955	Folates, riboflavin, vit. B_6_, vit. C, thiamine
*ADP-dependent*	5.605%	*vitamins*	26.641%	1.555%	0.232	0.054	0.249	0.944	Vit. B_6_, niacin, riboflavin, folates

R^2^ is a squared canonical correlation coefficient (canonical determination), which is relevant to overall variance between canonical variables. Total redundancy is relevant to the variance between the canonical variable for set II (RDA) and the variable describing whole blood platelet response to the agonization with AA, collagen or ADP; it shows the representation of a given canonical variable by the explanatory variables of set II. Extracted variance is the variance between a given canonical variable and the variables that build it up; it reflects how well a given canonical variable represents a given set of variables. Wilks’ lambda is a parameter reflecting the contribution of set II to the explaining of the variance of set I; the lower the Wilks’ lambda, the higher the contribution. Canonical correlation was calculated with the use of the bootstrap-boosted procedure of resampling with replacement (10,000 iterations). The RDAs of the following vitamins were included in set II (vitamins): vitamin A, retinol, β-carotene, vitamin E, thiamine, riboflavin, niacin, vitamin B_6_, vitamin C, folates, vitamin B_12_ and vitamin D.

## Data Availability

The data presented in this study are available on request from the corresponding author. The data are not publicly available due to ethical issues.
